# Prospective surveillance study of acute respiratory infections, influenza-like illness and seasonal influenza vaccine in a cohort of juvenile idiopathic arthritis patients

**DOI:** 10.1186/1546-0096-11-10

**Published:** 2013-03-07

**Authors:** Luciana M Carvalho, Flávia E de Paula, Rodrigo V D Silvestre, Luciana R Roberti, Eurico Arruda, Wyller A Mello, Virginia P L Ferriani

**Affiliations:** 1 Department of Pediatrics, School of Medicine of Ribeirão Preto, University of São Paulo, Avenida Bandeirantes 3900, S/N. Campus Universitário – Vila Monte Alegre, Ribeirão Preto 14049-900, Brazil; 2 Department of Cell Biology, School of Medicine of Ribeirão Preto, University of São Paulo, Avenida Bandeirantes 3900, S/N. Campus Universitário – Vila Monte Alegre, Ribeirão Preto 14049-900, Brazil; 3 Evandro Chagas Institute, WHO National Influenza Center, Rodovia BR-316 Km 7, Ananindeua 67030-000, Brazil

**Keywords:** Acute respiratory infections, Respiratory viruses, Influenza-like illness, Influenza vaccine, Juvenile idiopathic arthritis

## Abstract

**Background:**

Acute respiratory infections (ARI) are frequent in children and complications can occur in patients with chronic diseases. We evaluated the frequency and impact of ARI and influenza-like illness (ILI) episodes on disease activity, and the immunogenicity and safety of influenza vaccine in a cohort of juvenile idiopathic arthritis (JIA) patients.

**Methods:**

Surveillance of respiratory viruses was conducted in JIA patients during ARI season (March to August) in two consecutive years: 2007 (61 patients) and 2008 (63 patients). Patients with ARI or ILI had respiratory samples collected for virus detection by real time PCR. In 2008, 44 patients were immunized with influenza vaccine. JIA activity index (ACRPed30) was assessed during both surveillance periods. Influenza hemagglutination inhibition antibody titers were measured before and 30-40 days after vaccination.

**Results:**

During the study period 105 ARI episodes were reported and 26.6% of them were ILI. Of 33 samples collected, 60% were positive for at least one virus. Influenza and rhinovirus were the most frequently detected, in 30% of the samples. Of the 50 JIA flares observed, 20% were temporally associated to ARI. Influenza seroprotection rates were higher than 70% (91-100%) for all strains, and seroconversion rates exceeded 40% (74-93%). In general, response to influenza vaccine was not influenced by therapy or disease activity, but patients using anti-TNF alpha drugs presented lower seroconversion to H1N1 strain. No significant differences were found in *ACRPed30* after vaccination and no patient reported ILI for 6 months after vaccination.

**Conclusion:**

ARI episodes are relatively frequent in JIA patients and may have a role triggering JIA flares. Trivalent split influenza vaccine seems to be immunogenic and safe in JIA patients.

## Background

Acute respiratory infections (ARI) are prevalent worldwide and infections by influenza virus are particularly responsible for morbidity and mortality among children and other groups considered at high risk for complications from viral infections [1,2].

There have been no published studies on the prevalence of viral respiratory infections in juvenile idiopathic arthritis (JIA) patients and it is not known if these patients are at higher risk for severe influenza infections as compared to healthy children. Few studies have suggested ARI as trigger of JIA [3,4]. Available evidence suggests that influenza vaccination is safe and immunogenic in children with JIA. However, it is not known if influenza vaccination is actually protective against influenza-like illness (ILI) [5-12].

The objectives of this study were to evaluate the frequency of ARI and ILI in JIA patients and how they affect disease activity, and to assess immunogenicity, safety and efficacy of influenza vaccine in JIA patients treated with potentially immunosuppressive drugs, including anti-TNFα.

## Methods

### Study design and population

**Figure 1:**
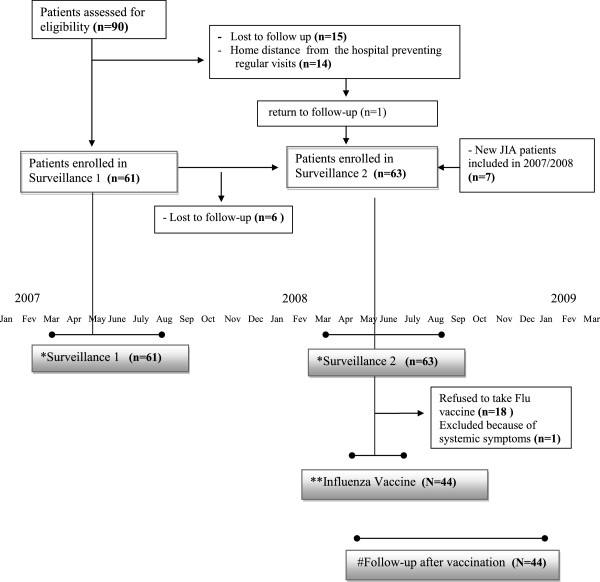
**Study time-table describing surveillance for viral respiratory infections, influenza vaccination and follow-up after vaccination period.** *Surveillance for upper respiratory viruses occurred during two subsequent years, from March to August: 2007- surveillance 1; and 2008 – surveillance 2. ** Influenza vaccine was offered between April and July 2008, during the surveillance 2 period. # Follow-up after vaccination: period of six months after the vaccine. n = number of patients included in each period.

We performed a longitudinal, prospective surveillance study of ARI and ILI episodes, and of influenza vaccination in a cohort of JIA patients, classified by the International *League Against Rheumatism* (ILAR) criteria, attending a tertiary Pediatric Rheumatology Clinic [13]. The surveillance occurred during the months of peak occurrence of ARI in two consecutive years: March through August 2007 (surveillance 1, SV1) and March through August 2008 (surveillance 2, SV2). Of the ninety eligible JIA patients, 61 participated of SV1 (15 patients lost follow up during SV1 and 14 patients were not able to attend regular visits due to home distance from the hospital). Sixty-three patients participated in SV2 period: 55 of the 61 patients who participated on SV1, 7 new JIA patients and 1 old patient that returned to follow up during this period (Figure 1).

Influenza vaccine was offered between April and July 2008 to all JIA patients attending the clinic that agreed to participate. Patients with at least one of the following criteria were excluded from vaccination: presence of systemic symptoms (fever, rash, vasculitis, hepatosplenomegaly, serositis); vaccination with any other vaccine in the previous month, allergy to components of influenza vaccine, and previous history of Guillain-Barre syndrome. Ten healthy children, between 3 and 18 years, received the influenza vaccine in order to evaluate the immunogenicity of that specific batch of the vaccine.

Routine clinical evaluations, including the *Pediatric American College of Rheumatology 30% (*ACRPed30) [14] were conducted during both surveillance periods prior to and at 30, 90 and 180 days after influenza vaccination.

JIA flares were defined as worsening by at least 40% in two or more of the six criteria in the ACRPed index, with simultaneous improvement of up to one criterion above 30% [15], and were considered as related to ARI or ILI if occurred up to 30 days after the ARI or ILI episodes. Disease activity was defined as presence of at least one joint with active arthritis or any systemic sign or symptom (fever, rash, vasculitis, hepatosplenomegaly, serositis) [16].

The study protocol was approved by the Ethics Committee of the Hospital das Clínicas da Faculdade de Medicina de Ribeirão Preto-USP (Process number 10542/2007) and the parents of all children and adolescents involved in the study gave informed consent for their participation.

### Surveillance for ARI and ILI episodes

ARI episodes were defined by the presence of one or more respiratory symptoms (rhinorrhea, sneezing, nasal obstruction, hoarseness, stridor, cough, dyspnea, tachypnea, chest retraction, wheezing or rhonchi). Cases of ILI were defined by the presence of fever plus at least one respiratory symptom (cough and/or sore throat) and one constitutional symptom (headache, malaise, myalgia, sweat or chills, or fatigue) [17,18]. Contacts with patients were made by telephone every two to three weeks and during their routine visits. Patients were also instructed to contact our medical staff in case of any respiratory symptom. Clinical assessment of disease activity, including laboratory tests, was carried out following the clinic routines. After the observation period, medical records were analyzed retrospectively to identify possible temporal associations, up to 30 days, between ARI/ILI episodes and signs of worsening of the underlying disease. During the two study periods, SV1 and SV2, patients with ARI and/or ILI had nasopharyngeal swabs collected for viral detection within the first 72 hours of symptoms, according to recommended procedures [19].

The attack rate was expressed as ARI episodes per 1,000 child-days at risk. A child-day was considered as 1 day of study for each child in the cohort. In calculating attack rate, only at-risk child-days were included in the denominator, with the removal of those days contained within an ARI and the 3 subsequent days [18,20]

### Detection of respiratory viruses

Nasopharyngeal swabs collected from both nostrils were immersed in viral transport medium (VTM) and immediately transported on ice to the Viral Pathogenesis Laboratory at the Virology Research Center, University of São Paulo, School of Medicine of Ribeirão Preto. Samples were then frozen at -70°C until being tested by real-time PCR for the presence of influenza A and B, respiratory syncytial virus (HRSV) A and B, metapneumovirus (HMPV), parainfluenza virus (HPIV) 1 and 3, bocavirus (HBov), adenovirus (HAdv), rhinovirus (HRV) and coronavirus OC43 e 229E (HCov).

Nucleic acids were extracted from 200 μL of sample using the QIAamp MinElute Virus Spin Kit (Qiagen, Hilden, Germany) following the manufacturer’s instructions. Detection of respiratory viruses was performed by real-time PCR (qPCR) using the TaqMan strategy (Applied Biosystems) with specific primers and probes (Additional file 1) [21-30] in a Thermocycler 7300 (Applied Biosystems). qPCR for the housekeeping β-actin gene was done in all samples as internal control. qPCR for HRSVA and B; HCoV OC43 and 229E; two sets of HRV serotypes; and HAdv and HBoV were done in duplex format, whereas single qPCR with only one pair of primers per reaction was done for HMPVA, HMPVB, influenza A, influenza B, HPIV1, HPIV3 and β-actin. For RNA viruses (HRV, influenza, HPIV, HRSV, HMPV and HCoV) reverse transcription was done prior to PCR using Multiscribe reverse transcriptase (Applied Biosystems) with 1 μg of RNA, primed with random hexamers, following the protocol proposed by the manufacturer detailed in the Additional file 1.

### Influenza vaccination

The trivalent split influenza vaccine containing A/Solomon Islands/3/2006, H1N1; A/Brisbane/10/2007, H3N2; B/Florida/4/2006, and B/Florida (Sanofi Pasteur SA/Butantan Institute), recommended for use in the Southern Hemisphere in 2008 [31] was given to patients and healthy children between March and May 2008. Patients and healthy children older than 9 years received one intramuscular dose of the vaccine (0.5 ml); and 3-8 and 1-3 year-old patients received two doses of 0.5 ml and 0.25 ml, respectively, 4 weeks apart, following established recommendations [32]. Local symptoms at the injection site and systemic symptoms were recorded.

### Influenza antibody titer determinations

Anti-influenza antibody titers were determined by hemagglutination inhibition (HI) prior to and 30-40 days post vaccination at the Respiratory Viruses Laboratory in Evandro Chagas Institute according to a standard WHO procedure [33].

Serum samples were stored at -70°C until testing, when they were treated with receptor-destroying enzyme and with turkey red blood cells to remove nonspecific agglutinins. Serum samples were then tested by HI for antibodies against the three antigens included in the vaccine. The working dilution (test dose) of each antigen contained four HI units in 25 μl. Test doses were diluted in phosphate-buffered saline and added to serial dilutions of antiserum (1:1 to 1:1024). The HI titer was determined as the reciprocal of the last serum dilution that completely inhibited red blood cell agglutination.

The titer of a serum not showing any inhibition at dilution 1:1 was assigned a value of 5.

A 4-fold rise in titers or a rise in titer from <20 to ≥40 was considered a significant response to vaccination. HI titers ≥40 are indicative of protection against infection. The geometric mean titers (GMT) were calculated using log-transformed titer values. A protection rate exceeding 70% of the vaccinated group, a seroconversion rate exceeding 40%, and a seroconversion factor exceeding 2.5 are considered as cut-off levels of vaccine immunogenicity, according to the guidelines of the European Committee for Proprietary Medicinal Products (CPMP) for the assessment of influenza vaccines in adults [34,35].

### Statistical methods

Statistical analysis was carried out using the SAS/STAT® system, version 9 (SAS Institute Inc., 2002).

To compare the frequency of episodes of ARI, ILI and worsening of indices of disease activity between periods of surveillance 1 and 2, and between vaccinated and unvaccinated patients the data were fitted in a zero-inflated Poisson model [36]. A multiple model for comparison of ARI and ILI episodes adjusted for other covariates: JIA type of onset (oligoarticular, polyarticular or systemic), gender, age (< 9 years old and ≥ 9 years old), disease activity and use of immunosuppressive therapy were performed.

The student *t* test was used to compare geometric means of HI titers. Calculations were performed on log-transformed values (base 10).

Fisher’s exact test was used to evaluate associations between the response rates to the 3 vaccine antigens and patient groups with different therapeutic regimens (methotrexate/leflunomide, TNF-α inhibitor), different age-groups (< 9 yrs and ≥ 9 yrs), JIA onset type (oligoarticular, polyarticular and systemic) and between patients with active disease. A logistic regression model adjusted to these covariates was performed for evaluate influence on seroconversion.

For comparison of variables related to disease activity before and after vaccination, the nonparametric Friedman test was used.

For all tests, a significance level of 5% was adopted.

## Results

### Frequency of ARI and ILI

Demographics and JIA clinical characteristics of the patients are summarized in Table 1.

**Table 1:** Characteristics of JIA patients participating in the two periods of surveillance for acute respiratory infections and in the phase of vaccination against influenza virus

	**SV 1**	**SV 2**	**Vaccination**
Patients (n)	61	63	44
Age, mean (years)	10.7	10.9	11.0
**ILAR categories n (%)**			
Oligoarthritis extended	8 (13.1)	12 (19)	9 (20.4)
Oligoarthritis persistent	12 (19.6)	11 (17.4)	11 (25)
Polyarticular RF -	20 (32.7)	19 (30.1)	12 (27.3)
Polyarticular RF+	4 (6.5)	5 (7.9)	4 (9)
Systemic	13 (21.3)	13 (20.6)	7 (16)
Undifferentiated arthritis	4 (6.5)	3 (4.8)	1 (2.3)
Use of immunosuppressant, DMARDS or anti-TNF drugs, n (%)	34 (55.7)	43 (68.2)	31(70)
Use of corticosteroids, n (%)	5 (8.2)	5 (7.9)	6 (13.6)
(mean daily dose corticosteroids: mg/kg/day)	0.45 (0.05-1)	0.34 (0.1-0.5)	0.3 (0.1-0.6)
Active disease **n (%)**	21 (34.4)	25 (39.6)	25(57)

Surveillance for respiratory infections: from March to August 2007- surveillance 1 (SV1) and from March to August 2008 – surveillance 2 (SV2). ***ILAR***: International League of Associations for Rheumatology, ***RF***: rheumatoid factor.

During the two surveillance periods, 105 ARI episodes were reported: 68 in 44 of 61 (72%) patients during the SV1 and 37 in 26 of 63 (41%) patients during the SV2. We evaluated the data of 9333 child-days in SV1 and 9639 child-days in SV2, 296 (3.1%) and 208 (2.1%) days respectively were contained within an ARI (ARI mean duration:5 days), and 204 and 111 days were subsequent not-at-risk days, leaving 8833 and 9320 days at-risk child-days (including the first day of each ARI) in SV1 and SV2 respectively. This gives an attack rate with 95% confidence interval (95% CI) of 7.6 (5.9-9.8) and 3.9 (2.8-5.5) ARI per 1000 child-days in SV1 and SV2 respectively. Twenty-eight of the 105 episodes (26.6%) were characterized as ILI: 23 during SV1 and 5 during SV2.

ARI and ILI episodes were significantly more frequent in SV1 than in SV2 (p < 0,01), even when adjusting for JIA type of onset, age (younger or older than 9 years), disease activity, use of immunosuppressives or DMARDs or administration of influenza vaccine.

There was no significant difference (p = 0.1) in the frequency of ARI episodes between vaccinated and unvaccinated patients. However, ILI episodes were significantly more common in unvaccinated patients (p = 0.02), although this difference was not maintained after adjusting for the other variables described above (p = 0.96). Of 33 naso-pharyngeal samples collected during the study, 20 (60.6%) were positive for at least one respiratory virus and viral co-infections were detected in 20% of the positive samples (Table 2).

**Table 2:** Episodes of acute respiratory infections and influenza-like illness and virus identification during the surveillance 1 and 2 periods in patients with JIA

	**SV 1**	**SV 2**	**p**
Patients (n)	61	63	
ARI episodes	68	37	<0.01*
ILI episodes (% of ARI)	23 (33,8)	5 (13,5)	<0.01*
Samples collected	26	7	
Positive samples - PCR (n)	Flu A (4)	Flu A (1)	
	Flu B (1)	HRV (1)	
	HRV (2)	HBov (1)	
	HPIV 1 (2)	HRSVA + HMPV (1)	
	HPIV 3 (1)		
	HAdv (2)		
	HRSVA (1)		
	HRV + HAdv (2)		
	HRV + HCov (1)		
N (%) of patients who received Flu Vaccine in the period	1 (1.5%)	44 (69.8%)	

*ARI*: Acute respiratory infection, *ILI*: Influenza-l*ike* illness*, Flu*: Influenza virus, *HRSV*: respiratory syncytial virus A and B, *HMPV*: metapneumovirus, *HPIV*: parainfluenza virus 1 and 3, *HBov*: bocavirus, *HAdv*: adenovirus, *HRV*: rhinovirus, *HCov*: coronavirus OC43 and 229E. ***SV1***: Surveillance 1, from March to August 2007. *SV2*: Surveillance 2, from March to August 2008, influenza vaccine was given in this period.* Poisson’s test.

Considering SV1 and SV2, a total of 28 ILI episodes were reported. Positive influenza samples were obtained in 5/14 ILI episodes of SV1 (35%) and in 1/7 ILI episodes during SV2 (14%), in a patient included prior to influenza vaccination. Although the most commonly detected virus during ILI episodes was influenza, detected in 7/28 (25%), HPIV (1 and 3) and HAdv were detected in one ILI episode each (Table 2).

All patients with ARI and ILI had favorable outcomes, except for one patient who developed acute otitis media and pneumonia after an ILI episode caused by HPIV infection.

### ARI and JIA flares

During the study 50 JIA flares or worsening of JIA activity parameters were observed in 44/70 patients included in at least one of two surveillance periods, and 10 of them (20%) were temporally associated with respiratory infection episodes (7 classified as ILI). In 8 of those episodes we could not identify any other triggering factor possibly associated with the flare (Table 3).

**Table 3:** **Temporal association between acute respiratory infections and reactivation or worsening of JIA activity parameters in 10 of 70 patients who participated in the epidemiological surveillance** 1 **and/or** 2 **for respiratory virus**

**Vírus detected**	**ARI**	**Onset type**	**Signs of disease worsening or flare**	**Temporal relationship**	**Therapeutic decision**
RSVA	NILI**	S	10% increase in the number of active joints, 50% worsening of the patient’s subjective evaluation	Concomitant	Increase the dose of cyclosporine
HAdv	ILI***	S	60% increase in the number of active joints and 200% worsening of the patient’s subjective evaluation	3 weeks	Intra-articular infection
HPIV1	ILI	S	Flare#	Concomitant	None. Symptoms improved with resolution of Flu-like symptoms
NC*	ILI	S	Flare	5 days	Increase the dose of methotrexate and oral prednisone course
HPIV3	ILI	S	Flare with systemic symptoms	7 days	Pulse of prednisone
HAdv	ILI	O	Flare	12 days	Restart methotrexate
NC	NILI	P	200% increase in number of active joints and 50% increase in ESR	7 days	Start nonsteroidal antiinflammatory
RSVA/HMPV	NILI	P	Flare	Concomitant	Increase prednisone dose
Flu A	ILI	P	Worsening of morning stiffness and joint effusion. Appearance of cysts on wrists.	Concomitant	Start etanercept and leflunomide
				Confounding factor: varicella 8 days before	
NC	ILI	S	Flare	1 week Confounding factor: methotrexate suspended 30 days before	Pulse of prednisone
					Restart methotrexate

*Flu*: Influenza virus A and B, *HRSV*: respiratory syncytial virus A and B, *HMPV*: metapneumovirus, *HPIV*: parainfluenza virus 1 and 3, *HBov*: bocavirus, *HAdv*: adenovirus, *HRV*: rhinovirus, *HCov*: coronavirus OC43 and 229E. *O*: oligoarticular, *P*: polyarticular, *S*: systemic *NC: not collected. **NILI: ARI not Flu-like. ***ILI: Flu-like illness # Flare: defined as worsening by 40% in two or more of the six ACRPed criteria with simultaneous improvement of up to one criterion above 30%. Surveillance 1: from March to August 2007. Surveillance 2: from March to August 2008, influenza vaccine was given in this period.

The possible factors associated with the 40 JIA flare episodes not related to respiratory infections were suspension or nonadhearance to medication in twelve episodes, and intercurrent infections in seven episodes: otitis, chickenpox, parotiditis (2), gastroenterocolitis (2) and infection sacroiliitis. In twelve-one episodes there were no identifiable causes for the flares.

There was no significant difference in the total number of flares related to gender, age (< 9 years old and ≥ 9 years old), JIA type of onset (oligoarticular, polyarticular or systemic), use of immunosuppressive therapy, period of surveillance (SV1 or SV2) or administration of influenza vaccine. Flares or worsening of JIA activity parameters associated with ARI were more frequently observed in patients with systemic JIA (5 of the 16 flares that occurred in this group) as compared to patients with polyarticular JIA (2/14 flares, p = 0.03).

### Response to influenza vaccine

Pre-vaccination geometric mean titers (GMT) with 95% confidence interval (95% CI) for H1N1, H3N2 and B/Florida in JIA patients were 13.3 (11-21.5), 12.4 (10.5-21) and 14.1 (12.2-23.3) respectively; and 43.4 (41.7-46.5), 33.2 (31.5-38.7) and 33.6 (31.9-37.6) in post vaccine period. Vaccine response of the patients and controls is described in Table 4.

**Table 4:** Strain-specific A/Salomon Islands/3/2006 (H1N1), A/Brisbane/10/2007 (H3N2) and B/Florida/4/2006 (B/Florida) hemagglutination inhibition titers, seroconversion factor, seroconversion rate, and seroprotection rate

	**H1N1**			**H3N2**			**B/Florida**		
	**Day 0**		**Day 30**	**Day 0**		**Day 30**	**Day 0**		**Day 30**
**Seroprotection rate**^**a **^**(%)**									
**JIA (44)**	21/43 (48.8)		44(100)	20/43 (46.5)		40(91)	20/43 (46.5)		42(95)
**Healthy children# (10)**	7(70)		10(100)	6(60)		8(80)	5(50)		10(100)
**Seroconversion rate**^**b **^**(%)**									
**JIA**			40/43(93)			32/43 (74.4)			31/40 (77.5)
**Healthy children**			7/7(100)			3/8 (37.5)			6/8 (75)
**Seroconversion factor**^**c**^									
**JIA**			16.5			5.2			7.2
**Healthy children**			9.8			3.0			4.0

To meet the Committee for Proprietary Medicinal Products (CPMP) guidelines each of the vaccine antigens must meet at least one of the following criteria: seroprotection rate exceeding 70%, seroconversion rate exceeding 40%, and seroconversion factor exceeding 2.5. ^a^ Seroprotection rate: the percentage of vaccine recipients with a serum HI titres of at least 1:40 after vaccination. ^b^Seroconversion rate: the percentage of vaccine recipients with an increase in serum HI titres by at least a factor of 4 after vaccination. ^c^ Seroconversion fator: the fold increase in serum geometric mean HI titres after vaccination.

Thirty-one of 44 patients (70%) who received influenza vaccine were using methotrexate or leflunomide, one patient was on cyclosporine, and five were receiving anti-TNFα drugs at the time of vaccination. Six patients were using corticosteroids at mean daily dose of 0.3 (0.1-0.6) mg/kg/day, with mean duration of treatment of 16.8 (10-24) months.

In general, response to influenza vaccine was not influenced by age, JIA type of onset, therapeutic regimens or disease activity. Patients on anti-TNFα drugs presented lower seroconversion (p = 0.03) and seroprotection (60%) responses to H1N1 strain, but the seroprotection above the cut-off levels to the other strains: H3N2 (100%) and B/Florida (80%).

The vaccine was considered safe. Pain at the injection site was described in 6/44 (13.6%) JIA patients and 1/10 (10%) healthy children; other local changes (redness and swelling or warmth) were reported by 2/44 (4.5%) of JIA patients and in 1/10 healthy children. Six patients developed cough and rinorrhea, without fever, during the first ten days after receiving the vaccine.

No significant differences were found in JIA activity index *(ACRPed30)* or doses of prednisone used by patients before and after 30, 90 and 180 days of vaccination (p = 0.22), Table 5. Methotrexate weekly dose was significantly lower 30 and 90 days after vaccination when compared to the pre vaccination dose (p = 0.03).

**Table 5:** **Parameters considered for JIA activity index *(ACRPed30) *of 44 JIA patients before and 30, 90 and 180 days after vaccination**

	**Pré Mean (SD)**	**30**	**90**	**180**	p*
**Number of joints with active arthritis**	1.4(2.4)	1.2(2.0)	1.1(1.7)	1.7(4.5)	0.39
**Number of joints with limited range of motion**	2.2(4.7)	2.2 (4.8)	2.3(5.1)	1.7(4.4)	0.96
**Physician’s global assessment of disease activity (0-10)**	1.3(1.4)	0.85(1.2)	0.92(1.4)	0.8(1.6)	0.65
**Parent/patient assessment of overall well being (0-10)**	1.3(1.8)	1.1(1.85)	1(1.5)	0.88(1.7)	0.76
**CHAQ**	0.2(0.5)	0.12(0.3)	0.05(0.2)	0.03(0.12)	0.29
**ESR (mm/hour)**	12.9(8.6)	14.6(10.2)	13(9.2)	12.1(9.3)	0.75

*ESR*, Erythrocyte sedimentation rate; *ACRPed30*, JIA activity index -*Pediatric American College of Rheumatology 30%*; *CHAQ*, Childhood Health Assessment Questionnaire.* Nonparametric Friedman test.

No patient reported ILI symptoms during the 6-month post-vaccine follow-up period.

## Discussion

As far as we know there are no published studies that evaluating the frequency and impact of ARI and ILI in JIA patients.

The observed attack rate of 7.6 and 3.9 ARI per 1000 child-days in 2007 and 2008, respectively, was similar to ARI rates previously reported in healthy children in the same age [37]. Viral agents were detected in 60% of 33 episodes sampled during the study, and influenza and HRV were the most frequently detected viruses. In studies conducted in tropical countries, when HRV was tested, it was the most frequently identified virus, and influenza detection ranged from 1 to 32% of positive samples. The differences in study design and virus detection assays may explain differences in frequencies of virus detection [17,18,38,39].

Viral dual infections were detected in 15% of positive samples in our study and HRV was identified in 75% of them. Co-infections were also detected in other studies, ranging from 9.5 to 81% of positive samples [39-41].

Evaluating only ILI episodes, corresponding to 26.6% of ARI episodes, influenza was the most frequently detected virus (25%), but HPIV (1 and 3) and HAdv were also detected. Other studies have also called attention to the fact that not only influenza is detected in ILI episodes. In Brazil, a study showed that 57% of ARI episodes were classified as ILI and influenza accounted for only 31% of them, with HRV being detected in 19.6% of ILI episodes [17]. Therefore, it is difficult to estimate influenza vaccine efficacy based only on the clinical analysis of ILI episodes.

The frequency and clinical pattern of ARI in patients with chronic disease have been poorly studied. Respiratory viruses were detected in 61 of 148 febrile episodes that occurred in 51 children with leukemia [42] and the frequency of ARI in 20 children with cystic fibrosis and 18 controls during a 6-month surveillance period was similar, but the morbidity of these infections was higher in patients with cystic fibrosis [43]. In our study, viral infections, including influenza infection, had a satisfactory outcome in JIA patients. Only one patient had acute otitis media and pneumonia associated with HPIV.

Despite the outcome of viral infections in our study being similar to the healthy population, the results suggest that viral ARI and ILI may be associated with JIA exacerbations or flares. Of 50 episodes of flares or worsening of disease activity observed during the study period, ten happened along with or within 3 weeks of a viral ARI. HRSV, HAdv, HPIV, HMPV and influenza were the viruses detected in association with these flares.

Few studies have addressed the possible relationships of viral ARI and JIA [3,4]. Recently, Toplak et all reported a JIA relapse after an ILI episode [12].

In the present study, 21 of the 50 JIA flares or worsening of JIA activity parameters (42%) were not temporally associated with any know triggers. In this way, it is impossible to determine whether there was a coincidental or causal relationship between ARI and JIA flares, but we can speculate that ARI may have had a role in 20% these worsening episodes. Another interesting finding of our study is that the majority of flares associated with viral infections occurred in patients with systemic onset JIA.

We have also assessed the safe, immunogenicity and effectiveness of influenza vaccine.

Prospective open-cohort studies suggest that influenza vaccination was safe [5-11], with no association with disease activity or autoantibodies induction [12]. In our study, patients were followed for a 6-month period after vaccination and no significant side occurred, including flares of the disease.

The immunogenicity of influenza vaccine in JIA patients has been previously looked at [5,6,8-12].

Malleson et al evaluated the immunogenicity of influenza vaccine in 34 children with chronic arthritis and in 13 healthy controls and no differences in antibody titers after vaccination were observed between children with chronic arthritis and controls, nor among patients treated with DMARDS and/or prednisone [6].

Kanadoudi-Tsakalidou et al evaluated 49 JIA patients, and also showed satisfactory vaccine immunogenicity and effectiveness with no ILI episode observed in a six-month period after the vaccine [5].

Ogimi et al evaluated 23 children with JIA and found no difference in seroconversion rates when compared gender, age, vaccine dosage, prednisolone dosage or use of other immunosuppressant by logistic regression analysis. That study was the first to evaluate influenza vaccine seroconversion in one patient with JIA under treatment with anti-TNF [8].

The immunogenicity and safety of epidemic influenza A H1N1/2009 vaccine were studied in 237 patients with autoimmune rheumatic disease (ARD), 93 JIA, and in 91 healthy controls. The authors demonstrated a reduced immune response to this vaccine in ARD patients when compared to controls and found glucocorticoid use as the major factor for decreased antibody production [9].

The immunogenicity of influenza vaccine was recently investigated in 27 patients with systemic-onset juvenile idiopathic arthritis receiving tocilizumab, an anti-interleukin-6 receptor antibody. The authors observed that high-dose prednisolone, but not tocilizumab, impaired the production of influenza antibodies in these patients [11].

Toplak et all recently showed adequate response to influenza vaccine in JIA patients, except in a subgroup of 4 children receiving anti-TNF therapy, and observed no difference in the rate of influenza infection between 31 JIA patients and 14 healthy children followed for 6 months after vaccination [12].

In our study the immunogenicity of influenza vaccine was found to be satisfactory. We found no influence of age, JIA type of onset, activity of disease, use of prednisolone or methotrexate in seroconversion rates. Only the subgroup of patients using anti TNF alpha showed lower seroconversion rate for the H1N1 strain. As the sample size in the present study is small, it was not possible to assess the adjusted odds ratio of using anti-TNF alpha in relation to other variables. A study with children with inflammatory bowel disease showed that patients receiving anti-TNF therapy were less likely to be seroprotected against B strain [44].

Dell’Era et al recently evaluated if the administration of the more immunogenic adjuvanted influenza vaccine (MF59-adjuvanted seasonal vaccine) could overcome the problem of the possibly impaired antibody production in 60 JIA patients (30 treated with etarnecept). The safety and tolerability of the vaccine were satisfactory, but the results of this study indicate a reduced immune response to this vaccine in JIA children and adolescents treated with etanercept in comparison with those with DMARDs and healthy controls [10].

This was the first study that assessed the effectiveness of influenza vaccine thorough the surveillance of vaccinated and unvaccinated patients during the influenza seasonal periods and there was no significant difference in the frequency of ARI episodes after the vaccination. Episodes classified as ILI were more common in unvaccinated patients, but this difference was not maintained after adjusting for other covariates such as surveillance period, age, immunosuppressant use and active disease. We believe that the failure to maintain significance of IL1 episodes in vaccinated patients is related to the small sample size since we did not identify any specific covariate responsible to this failure when we performed the multiple model analysis. Thus, it is not possible to prove that the vaccine was really effective against influenza infections, as the pattern or seasonality of influenza infections seemed to be different between the two surveillance periods. However, it is important to state that during the follow-up period of six months after vaccination, none of the vaccinated patients had ILI episodes.

## Conclusion

Infections caused by common respiratory viruses may be associated with JIA flares, a hypothesis to be addressed in further, larger epidemiological studies. Trivalent split influenza vaccine seems to be immunogenic, safe and effective in children and adolescents with JIA, despite the use of immunosuppressive agents or activity of the disease. Studies of larger numbers of patients treated with TNFα blockers are necessary in order to verify whether a lower vaccine response occurs in this group of patients.

## Competing interest

There are no competing interest for the authors above and this work.

## Authors’ contributions

LC, EA, VF contributed to the design of the study. LC and VF participated in the interviews and chart revision. LC and LR performed the data and study samples collection, analysis and interpretation. FP carried out the real-time PCR for viral detection and analysis and interpretation of the data. RS, WM and LC carried out the hemagglutination inhibition assay for detecting anti-Flu antibody, analysis and interpretation of the data. LC wrote the manuscript and EA and VF helped in revisions and suggestion. All authors read and approved the final manuscript.

## Supplementary Material

Additional file 1 ** Detection of Respiratory viruses. Table S1**. Specific primers and probes used for respiratory viruses’ detection by real-time PCR (qPCR). Click here for file
