# Supplementary material for: Prospective surveillance study of acute respiratory infections, influenza-like illness and seasonal influenza vaccine in a cohort of juvenile idiopathic arthritis patients
Source: Pediatr Rheumatol Online J. 2013 Mar 7;11:10. doi: 10.1186/1546-0096-11-10 (PMC3602114; doi:10.1186/1546-0096-11-10)
Supplement: Additional file 1 — Detection of Respiratory viruses. Table S1. Specific primers and probes used for respiratory viruses’ detection by real-time PCR (qPCR). [file 1546-0096-11-10-S1.docx]

**Text: Detection of Respiratory viruses**

Duplex qPCRs were done in a final volume of 15 µL with 3 µl of cDNA, 10µM of forward and reverse primers, 5 µM of probe, 7.5 µl of TaqMan master mix (Applied Biosystems), with the following parameters: 95°C for 10 minutes, followed by 45 cycles of 95°C for 15 seconds and 60°C for 1 minute. The qPCRs with a single pair of primers were done in a final volume of 10 µL with 3 µl of cDNA, 10µM of forward and reverse primers, 5 µM of probe, 5 µl of TaqMan master mix (Applied Biosystems), with the following parameters: 95°C for 10 minutes, followed by 45 cycles of 95°C for 15 seconds, and 60°C for 1 minute, except for HMPV A and B, when the parameters used were 95°C for 10 minutes, followed by 45 cycles of 95°C for 15 seconds, 55°C for 30 seconds, and 60°C for 1 minute. For DNA viruses qPCR was done with 3 µl of extracted DNA, in a final volume of 15µL, containing 10µM of forward and reverse primers, 5µM of probe and 7.5 uL of TaqMan master mix (Applied Biosystems), using the same cycling parameters as for the RNA viruses.

**Table S1:** Specific primers and probes used for respiratory viruses’ detection by real-time PCR (qPCR)

| **VÍRUS** | **PRIMER** | **5'-3' sequence** | **Target gene** | **references** |
| --- | --- | --- | --- | --- |
|  | A21 | GCTCTTAGCAAAGTCAAGTTGAATGA |  |  |
| **HRSVA** | A102 | TGCTCCGTTGCATGGTGTATT | Nucleoprotein | [[1](#_ENREF_1)] |
|  | APB48 | **FAM**-ACACTCAACAAAGATCAACTTCTGTC-**TAMRA** |  |  |
|  | B17 | GATGGCTCTTAGCAAAGTCAAGTTAA |  |  |
| **HRSVB** | B120 | TGTCAATATTATCTCCTGTACTACGTTGAA | Nucleoprotein | [[1](#_ENREF_1)] |
|  | BPB45 | **JOE**-TGATACATTAAATAAGGATCAGCTGCTGTCATCCA**-TAMRA** |  |  |
|  | INFA-1 | GGACTGCAGCGTAGACGCTT |  |  |
| **Flu A** | INFA-2 | CATCCTGTTGTATATGAGGCCCAT | Matrix protein | [[2](#_ENREF_2)] |
|  | INFA-3 | CATTCTGTTGTATATGAGGCCCAT |  |  |
|  | INFA PROBE | **FAM**-CTCAGTTATTCTGCTGGTGCACTTGCCA**-TAMRA** |  |  |
|  | INFB-1 | AAATACGGTGGATTAAATAAAAGCAA |  |  |
| **Flu B** | INFB-2 | CCAGCAATAGCTCCGAAGAAA | Hemaglutinin | [[2](#_ENREF_2)] |
|  | INFB PROBE | **JOE**-CACCCATATTGGGCAATTTCCTATGGC-**TAMRA** |  |  |
|  | HRV forward | GCACTTCTGTTTCCCC |  |  |
| **HRV** | HRV reverse | GGCAGCCACGCAGGCT | 5'UTR* | [[3](#_ENREF_3)] |
|  | HRV1 probe | **FAM**-AGCCTCATCTGCCAGGTCTA-**MGB** |  |  |
|  | HRV2 probe | **VIC**-AGCCTCATCGACCAAACTA-**MGB** |  |  |
|  | HMPVA forward | GCC GTT AGC TTC AGT CAA TTC AA |  |  |
|  | HMPVA reverse | TCC AGC ATT GTC TGA AAA TTG C | Fusion protein |  |
| **HMPV** | HMPVA probe | **6FAM**-CAA CAT TTA GAA ACC TTC T-**MGBNFQ** |  | [[4](#_ENREF_4)] |
|  | HMPVB forward | GCT GTC AGC TTC AGT CAA TTC AA |  |  |
|  | HMPVB reverse | GTT ATC CCT GCA TTG TCT GAA AAC T | Fusion protein |  |
|  | HMPVB probe | **6FAM**-CGC ACA ACA TTT AGG AAT CTT CT-**MGBNFQ** |  |  |
|  | Adeno1 | GCCACGGTGGGGTTTCTAAACTT |  |  |
| **HAdv** | Adeno2 | GCCCCAGTGGTCTTACATGCACATC | Hexon gene | [[5](#_ENREF_5)] |
|  | Adeno Sonda | **FAM**-TGCACCAGACCCGGGCTCAGGTACTCCGA-**TAMRA** |  |  |
|  | HBov forward | GCACAGCCACGTGACGAA |  |  |
| **HBov** | HBov reverse | TGGACTCCCTTTTCTTTTGTAGGA | NP1 | [[6](#_ENREF_6)] |
|  | HBov sonda | **JOE**-TGAGCTCAGGGAATATGAAAGACAAGCATCG-**TAMRA** |  |  |
|  | Para1 forward | ACAGATGAAATTTTCAAGTGCTACTTTAGT |  |  |
| **HPIV 1** | Para1 reverse | GCCTCTTTTAATGCCATATTATCATTAGA | Polymerase | [[7](#_ENREF_7)] |
|  | Para1 probe | **FAM**-ATGGTAATAAATCGACTCGCT-**MGB** |  |  |
|  | Para3 forward | CTC GAG GTT GTC AGG ATA TAG |  |  |
| **HPIV 3** | Para3 reverse | CTT GGG AGT TGA ACA CAG TT | Hemagglutinine Neuraminidase | [[8](#_ENREF_8)] |
|  | Para3 sonda | **FAM**-AAT AAC TGT AAA CTC AGA CTT GGT ACC TGA CTT-**TAMRA** |  |  |
|  | Corona F3 | TGGCGGGTGGGATAATATGT |  |  |
| **HCov-229E** | Corona R3 | GAGGGCATAGCTCTATCACACTTAGG | Polymerase 1b gene | [[9](#_ENREF_9)] |
|  | Corona P2 | **VIC**-ATAGTCCCATCCCATCAA-**MGB** |  |  |
|  | Corona F-OC | CCTTATTAAAGATGTTGACAATCCTGTAC |  |  |
| **HCov-OC43** | Corona R-OC | AATACGTAGTAGGTTTGGCATAGCAC | Polymerase 1b gene | [[9](#_ENREF_9)] |
|  | Corona P-OC | **FAM**-CACACTTAGGATAGTCCCA-**MGB** |  |  |
|  | β-Actina For. | CCCAGCCATGTACGTTGCTA |  |  |
| **β-Actin** | β-Actina Rev. | TCACCGGAGTCCATCACGAT | β-actin | [[10](#_ENREF_10)] |
|  | β-Actina Sonda | **VIC**-ACGCCTCTGGCCGTACCACTGG-**TAMRA** |  |  |

*5´UTR: untranslated region. Flu: Influenza virus A and B, HRSV: respiratory syncytial virus A and B, HMPV: metapneumovirus, HPIV: parainfluenza virus 1 and 3, HBov: bocavirus, HAdv: adenovirus, HRV: rhinovirus, HCov: coronavirus OC43 e 229E.

**Table References**

1 Hu A, Colella M, Tam JS, Rappaport R, Cheng SM. Simultaneous detection, subgrouping, and quantitation of respiratory syncytial virus A and B by real-time PCR. J Clin Microbiol 2003;41(1):149-54.

2 van Elden LJ, Nijhuis M, Schipper P, Schuurman R, van Loon AM. Simultaneous detection of influenza viruses A and B using real-time quantitative PCR. J Clin Microbiol 2001;39(1):196-200.

3 Deffernez C, Wunderli W, Thomas Y, Yerly S, Perrin L, Kaiser L. Amplicon sequencing and improved detection of human rhinovirus in respiratory samples. J Clin Microbiol 2004;42(7):3212-8.

4 Kuypers J, Wright N, Corey L, Morrow R. Detection and quantification of human metapneumovirus in pediatric specimens by real-time RT-PCR. J Clin Virol 2005;33(4):299-305.

5 Heim A, Ebnet C, Harste G, Pring-Akerblom P. Rapid and quantitative detection of human adenovirus DNA by real-time PCR. J Med Virol 2003;70(2):228-39.

6 Neske F, Blessing K, Tollmann F, et al. Real-time PCR for diagnosis of human bocavirus infections and phylogenetic analysis. J Clin Microbiol 2007;45(7):2116-22.

7 Kuypers J, Wright N, Ferrenberg J, et al. Comparison of real-time PCR assays with fluorescent-antibody assays for diagnosis of respiratory virus infections in children. J Clin Microbiol 2006;44(7):2382-8.

8 Garbino J, Gerbase MW, Wunderli W, et al. Lower respiratory viral illnesses: improved diagnosis by molecular methods and clinical impact. Am J Respir Crit Care Med 2004;170(11):1197-203.

9 Kuypers J, Martin ET, Heugel J, Wright N, Morrow R, Englund JA. Clinical disease in children associated with newly described coronavirus subtypes. Pediatrics 2007;119(1):e70-6.

10 Nystrom K, Biller M, Grahn A, Lindh M, Larson G, Olofsson S. Real time PCR for monitoring regulation of host gene expression in herpes simplex virus type 1-infected human diploid cells. J Virol Methods 2004;118(2):83-94.
